# Factors Associated With Quality-of-Dying-and-Death Classes Among Critically Ill Patients

**DOI:** 10.1001/jamanetworkopen.2024.20388

**Published:** 2024-07-01

**Authors:** Fur-Hsing Wen, Wen-Chi Chou, Chung-Chi Huang, Tsung-Hui Hu, Li-Pang Chuang, Siew Tzuh Tang

**Affiliations:** 1 Department of International Business, Soochow University, Taiwan, R.O.C.; 2 Division of Hematology-Oncology, Chang Gung Memorial Hospital at Linkou, Tao-Yuan, Taiwan, R.O.C.; 3 College of Medicine, Chang Gung University, Tao-Yuan, Taiwan, R.O.C.; 4 Department of Internal Medicine, Division of Pulmonary and Critical Care Medicine, Chang Gung Memorial Hospital at Linkou, Tao-Yuan, Taiwan, R.O.C.; 5 Department of Respiratory Therapy, Chang Gung University, Tao-Yuan, Taiwan, R.O.C.; 6 Department of Internal Medicine, Division of Hepato-Gastroenterology, Chang Gung Memorial Hospital at Kaohsiung, Kaohsiung, Taiwan, R.O.C.; 7 Department of Nursing, Chang Gung Memorial Hospital at Kaohsiung, R.O.C.; 8 School of Nursing, Medical College, Chang Gung University, Tao-Yuan, Taiwan, R.O.C.; 9 Department of Nursing, Chang Gung University of Science and Technology, Tao-Yuan, Taiwan, R.O.C.

## Abstract

**Importance:**

Improving end-of-life care in the intensive care unit (ICU) is a priority, but clinically modifiable factors of quality of dying and death (QODD) are seldom identified.

**Objectives:**

To comprehensively identify factors associated with QODD classes of dying ICU patients, emphasizing clinically modifiable factors based on the integrative framework of factors associated with for bereavement outcomes.

**Design, Setting, and Participants:**

This observational cohort study was conducted at medical ICUs of 2 Taiwanese medical centers from January 2018 to March 2020 with follow-up through December 2022. Eligible participants included primary family surrogates responsible for decision making for critically ill ICU patients at high risk of death (Acute Physiology and Chronic Health Evaluation II score >20) but who survived more than 3 days after ICU admission. Data analysis was conducted from July to September 2023.

**Main Outcomes and Measures:**

QODD was measured by the 23-item ICU-QODD questionnaire. Factors associated with patient membership in 4 previously determined QODD classes (high, moderate, poor to uncertain, and worst) were examined using a 3-step approach for latent class modeling with the high QODD class as the reference category.

**Results:**

A total of 309 family surrogates (mean [SD] age, 49.83 [12.55] years; 184 women [59.5%] and 125 men [40.5%]) were included in the study. Of all surrogates, 91 (29.4%) were the patients’ spouse and 66 (53.7%) were the patients’ adult child. Patient demographics were not associated with QODD class. Two family demographics (age and gender), relationship with the patient (spousal or adult-child), and length of ICU stay were associated with QODD classes. Patients of surrogates perceiving greater social support were less likely to be in the poor to uncertain (adjusted odds ratio [aOR], 0.89; 95% CI, 0.83-0.94) and worst (aOR, 0.92; 95% CI, 0.87-0.96) QODD classes. Family meetings were associated with the poor to uncertain QODD class (aOR, 8.61; 95% CI, 2.49-29.74) and worst QODD class (aOR, 7.28; 95% CI, 1.37-38.71). Death with cardiopulmonary resuscitation was associated with the worst QODD class (aOR, 7.51; 95% CI, 1.12-50.25). Family presence at patient death was uniformly negatively associated with the moderate QODD class (aOR, 0.16; 95% CI, 0.05-0.54), poor to uncertain QODD class (aOR, 0.21; 95% CI, 0.05-0.82), and worst QODD class (aOR, 0.08; 95% CI, 0.02-0.38). Higher family satisfaction with ICU care was negatively associated with the poor to uncertain QODD class (aOR, 0.93; 95% CI, 0.87-0.98) and worst QODD class (aOR, 0.86; 95% CI, 0.81-0.92).

**Conclusions and Relevance:**

In this cohort study of critically ill patients and their family surrogates, modifiable end-of-life ICU-care characteristics played a more significant role in associations with patient QODD class than did immutable family demographics, preexisting family health conditions, patient demographics, and patient clinical characteristics, thereby illuminating actionable opportunities to improve end-of-life ICU care.

## Key Points

**Question:**

What factors are associated with surrogate-assessed quality-of-dying-and-death (QODD) classes (high, moderate, poor to uncertain, and worst) for critically ill patients in the intensive care unit (ICU)?

**Findings:**

This cohort study of 309 family caregiver surrogates found that social support for family surrogates, family presence at death, and family satisfaction with ICU care were negatively associated with the moderate, poor to uncertain, and worst QODD classes. Death with cardiopulmonary resuscitation was associated with the worst QODD class, while family meetings were associated with the poor to uncertain and worst QODD classes.

**Meaning:**

Modifiable end-of-life ICU-care characteristics were more commonly associated with ICU patients’ QODD classes than immutable family demographics, patient demographics, and clinical characteristics, illuminating opportunities to improve end-of-life ICU care.

## Teaser

This cohort study assesses factors associated with family surrogates in Taiwan and their critically ill loved one’s categorization into 1 of 4 response classes based on the Quality of Dying and Death Questionnaire.

## Introduction

Increasing use of intensive care units (ICUs)^1^ and high-intensity ICU treatments^2^ at end of life (EOL) escalates health care costs^3^ and personal and family out-of-pocket spending^4^ but may not reflect patient preferences.^5^ Quality of EOL care in ICUs is improving but still poor.^6,7,8^ Improving EOL care quality has been nationally prioritized^9,10^ to promote value-concordant EOL care and mitigate unsustainable, skyrocketing costs in critical care.^11^

Improving EOL care and facilitating patient-^12^ and family^13^-centered care in ICUs requires measurement of EOL care quality alongside evaluation of factors, especially from patient and family perspectives, of high-quality dying and death.^14,15^ The Quality of Dying and Death (QODD) questionnaire is widely used and recognized as the best validated measure of quality of dying and death among a variety of populations.^16,17,18^ The few studies using the QODD with ICU patients^12,19,20,21^ have been conducted on family members for feasibility^14^ and reflect family-centered values in improving care.^15^ Through family member evaluation of the QODD frequency component, we previously identified 4 distinct QODD classes (high, moderate, poor to uncertain, and worst) characterized by patients’ physical symptom control, emotional preparedness for death, and amount of life-sustaining treatments (LSTs) received,^22^ reflecting the core domains for quality of death.^23^

However, of the few studies that investigated factors associated with QODD scores, all but 2 studies,^12,21^ were on patients with cancer or patients receiving palliative care.^24,25,26,27,28^ Most focused on patient^12,21,24,25,26,27^ or family^12,25,26,27^ demographics, patient clinical characteristics,^12,24,25,26^ family preexisting psychological distress,^28^ and patient-family relationship,^12,26,27^ but seldomly focused on modifiable EOL care.^12,24^ This lack of information on modifiable EOL care factors that differentiate levels of perceived quality of dying and death for critically ill patients represents a substantial gap in current knowledge. Therefore, we aimed to comprehensively identify factors for the 4 previously determined QODD classes, emphasizing clinically modifiable factors based on the integrative framework of factors associated with bereavement outcomes.^29^

## Methods

### Study Design, Setting, and Participants

This cohort study is part of a longitudinal, observational study on associations of quality of EOL ICU care measured by process-based indicators,^30^ family perception,^31^ and bereaved family surrogates’ psychological distress, including symptoms of anxiety,^32^ depression,^32^ and posttraumatic stress disorder.^33^ Adult family surrogates responsible for making EOL care decisions for critically ill ICU patients at high risk of death (APACHE II scores >20) but survived more than 3 days after ICU admission were consecutively recruited at 7 level III medical ICUs of 2 academically affiliated hospitals in northern and southwestern Taiwan from January 2018 to March 2020 and followed up through December 2022. The Chang Gung Medical Foundation institutional review board approved this study. Each surrogate signed written informed consent for participation and review of patient medical record. This study was reported according to the Strengthening the Reporting of Observational Studies in Epidemiology (STROBE) reporting guideline for cohort studies.

### Data Collection

Participant demographics and preexisting comorbidities were recorded at enrollment. Patient disease severity and patient-level process-based quality measures over the ICU stay were abstracted from medical records by trained research assistants.^30^ Surrogates were phone interviewed to assess their perception of their loved one’s quality of dying and death, their satisfaction with ICU care, and their perceived social support at 1-month after loss.

### Outcome Variable: Distinct QODD Classes

Quality of dying and death in ICUs was measured by the 23-item ICU-QODD. The ICU-QODD retains items most relevant for evaluating critically ill patients’ QODD and assesses 6 domains: symptoms and personal care, preparation for death, family concerns, treatment received, whole person concerns, and moment of death. Using the frequency component of each item and retaining unknown responses, we previously identified 4 distinct QODD latent classes: high, moderate, poor to uncertain, and worst. Classes were characterized by physical symptom control, emotional preparedness for death (eg, feeling at peace with dying, unafraid of dying, keeping one’s dignity and self-respect, clearing up bad feelings, concerns of imposing strains on family, saying goodbye to family, and having control of events), and amount of LSTs received.^22^ Unique characteristics of each QODD class are in Table 1. Although classes showed decremental differences (from high to worst) in adequate physical symptom control and incremental differences in amount of LSTs received, endorsement of each item of emotional preparedness for death did not uniformly decrease across the 4 QODD classes, especially for the poor to uncertain QODD class. Details on identification of the 4 QODD latent classes by latent class analysis have been described^22^ (eMethods in Supplement 1).

**Table 1.** Comparisons of QODD Characteristics Across the 4 Identified QODD Classes

Indicators	QODD class				
	High	Moderate	Poor to uncertain	Worst	
Physical symptom control	Optimal	Adequate	Inadequate		Poorest
Emotional preparedness for death	Moderate to sufficient	Moderate to sufficient	Insufficient or uncertain		Insufficient to moderate
Religious support	Ample	Ample	Adequate		Limited
Family support	Strong	Moderate to strong	Moderate to strong		Poor to moderate
Life-sustaining treatments received	Few	Least	Some		Substantial
Funeral arrangement in order	No	Yes	Yes		Yes

Abbreviation: QODD, quality of dying and death.

### Independent Variables

Factors of QODD class were selected across (1) intrapersonal risk factors, (2) interpersonal risk factors, (3) death-related factors, and (4) death circumstances based on the integrative framework of factors associated with bereavement outcomes,^29^ omitting appraisal and coping. Intrapersonal risk factors included family surrogates’ sociodemographics (gender, age, marital status [married vs unmarried], and education level [≤ high school or >high school]) and predisposing vulnerabilities such as financial hardship (self-reported financial insufficiency to make ends meet [yes or no]) and preexisting health conditions (emergency department visits, hospitalizations, and pain or mood medication use in the year before the patients’ critical illness).^34^

Interpersonal risk factors were measured by type of loss (ie, the surrogate-patient relationship [spouse, adult child, and other]) and surrogates’ perceived social support by the Medical Outcomes Study Social Support Survey,^35^ which assesses positive social interaction as well as emotional, informational, tangible, and affectionate support. Total scores are computed to on a scale from 0 to 100; higher scores indicate better perceived social support.

Death-related factors referred to decedents’ demographics (gender and age) and clinical characteristics. Clinical characteristics included diagnosis (cancer vs others), disease severity (Acute Physiology and Chronic Health Evaluation II [APACHE II] scores and ICU length of stay).

Death circumstances were indicated by medical record–derived, process-based indicators of high-quality EOL care in ICUs and family surrogates’ satisfaction with EOL ICU care. Selected process-based indicators of high-quality EOL care included prognostic communication, palliative care provided, family meetings conducted, social worker involvement, a do-not-resuscitate order issued before patient death, death with cardiopulmonary resuscitation (CPR), withdrawal of any LSTs, and family presence at death.^30,36^ The processes for ensuring interrater reliability in abstracted data for each quality indicator from medical records were established and reported.^30^

Family satisfaction with care was measured with the validated^37^ Family Satisfaction in the Intensive Care Unit questionnaire (FS-ICU).^38^ The 14-item FS-ICU Care subscale assesses satisfaction with (1) quality, continuity, and accessibility of care and (2) ICU and waiting room atmosphere. The 10-item FS-ICU Decision-Making subscale measures family satisfaction with the information and support provided during critical-care decision-making. Original item responses (1-5 Likert scale) were rescaled from 0 (least satisfied) to 100 (most satisfied). Mean scores of each subscale were calculated.

### Statistical Analysis

Factors associated with critically ill patients’ QODD class were identified by the 3-step approach in Latent GOLD statistical software version 5.1 (Statistical Innovations).^39^ In the first step, a latent class model was built based on the frequency component of each QODD item retaining unknown responses. Second, participants were assigned to QODD classes. Third, associations of proposed factors with QODD classes were modeled using the high QODD class as reference, accounting for classification error from assigning participants to classes.^39^ Exponentiating the regression coefficient for each independent variable resulted in adjusted odds ratios (aORs) with 95% CIs. The threshold for statistical significance was a 2-sided *P* < .05. Data analysis was conducted from July to September 2023.

## Results

### Participant Characteristics and Patient QODD Classes Assessed at First Month Postloss

Among the 353 ICU decedents, 309 family caregiver surrogates (mean [SD] age, 49.83 [12.55] years; 184 women [59.5%] and 125 men [40.5%]) participated in bereavement surveys (87.5%) and constituted the sample. Participants were predominantly married (233 caregivers [75.4%]). Of all caregivers, 91 (29.4%) were the patients’ spouse and 66 (53.7%) were the patients’ adult child (Table 2). Patients differed by QODD class; 130 patients (42.1%) were categorized as high, 59 (19.1%) as moderate, 67 (21.7%) as poor to uncertain, and 53 (17.2%) as worst.

**Table 2.** Characteristics of Participants

Characteristics	Participants No. (%) (N = 309)
Patient characteristics	
Age, mean (SD) [range], y	66.51 (14.18) [22-101]
Gender	
Men	196 (63.4)
Women	113 (36.6)
Primary disease	
Cancer	156 (50.5)
Pulmonary	20 (6.5)
Cardiovascular	14 (4.5)
Kidney	15 (4.9)
Other	104 (33.6)
Acute symptom or problem at admission	
Respiratory failure or distress	160 (51.8)
Infection	87 (28.2)
Shock	24 (7.8)
Cardiac arrest	12 (3.9)
Other^a^	26 (8.4)
Other chronic diseases	
Yes	262 (84.8)
No	47 (15.2)
Quality-of-dying-and-death class	
High	130 (42.1)
Moderate	59 (19.1)
Poor to uncertain	67 (21.7)
Worst	53 (17.2)
APACHE II score, mean (SD) [range]^b^	28.46 (5.38) [8-45]
Length of intensive care unit stay, mean (SD) [range], d	21.22 (15.17) [3-106]
Time from enrollment to death, mean (SD) [range], d	7.26 (8.36) [1-58]
Family caregiver characteristics	
Age mean (SD) [range], y	49.83 (12.55) [21-80]
Gender	
Male	125 (40.5)
Female	184 (59.5)
Relationship with the patient	
Spouse	91 (29.4)
Adult child	166 (53.7)
Other	52 (16.8)
Marital status	
Not married	76 (24.6)
Married	233 (75.4)
Educational level	
>High school	154 (49.8)
≤High school	155 (50.5)
Financial hardship	
No	259 (83.8)
Yes	50 (16.2)

Abbreviation: APACHE II, Acute Physiology and Chronic Health Evaluation.

^a^ Other diseases included diabetes, peptic ulcer, gastrointestinal disorders, chronic hepatitis, liver cirrhosis, dementia, AIDS, and systemic lupus erythematosus.

^b^ Measure of disease severity at enrollment.

### Factors of QODD Class Membership

QODD class membership was associated with 2 intrapersonal risk factors: gender and age (Table 3 and the eFigure in Supplement 1). In reference to the high QODD class, the older the surrogate, the lower the likelihood of patient membership in the worst QODD class (aOR, 0.92; 95% CI, 0.85-1.00). Family caregivers who were men were less likely to report patient membership in the poor to uncertain QODD class than women caregivers (aOR, 0.36; 95% CI, 0.13-0.99).

**Table 3.** Factors Associated With Quality-of-Dying-and-Death Classes Perceived by Bereaved Family Surrogates of Critically Ill Patients in ICUs

Independent variables	Quality-of-dying-and-death class^a^					
	Moderate		Poor to uncertain		Worst	
	aOR (95% CI)	*P* value	aOR (95% CI)	*P* value	aOR (95% CI)	*P* value
Intrapersonal risk factors (family demographics and preexisting mental health or medical conditions)						
Age	0.96 (0.91-1.01)	.09	1.02 (0.95-1.09)	.60	0.92 (0.85-1.00)	.05
Gender (men vs women)	0.65 (0.25-1.69)	.38	0.36 (0.13-0.99)	.05	0.59 (0.16-2.15)	.43
Educational attainment (≤high school vs >high school)	0.90 (0.24-3.39)	.88	1.22 (0.26-5.73)	.80	2.26 (0.28-17.99)	.44
Marital status (married vs unmarried)	1.47 (0.49-4.39)	.49	2.81 (0.77-10.33)	.12	4.67 (0.96-22.59)	.06
Financial hardship (yes vs no)	0.59 (0.16-2.13)	.42	0.53 (0.08-3.32)	.50	0.53 (0.08-3.71)	.52
Hospitalizations (yes vs no)^b^	0.53 (0.02-12.45)	.70	0.39 (0.01-17.33)	.63	NA	NA
Emergency department visits (yes vs no)^b^	0.18 (0.01-2.18)	.18	0.45 (0.04-5.71)	.54	2.39 (0.19-30.10)	.50
Use of pain medications (yes vs no)^b^	0.86 (0.24-3.08)	.81	1.66 (0.25-11.10)	.60	2.81 (0.36-21.98)	.33
Use of medications for mood disturbance (yes vs no)^b^	1.10 (0.09-12.86)	.94	3.15 (0.32-31.29)	.33	3.05 (0.20-46.53)	.42
Interpersonal risk factors						
Perceived social support	1.00 (0.97-1.04)	.87	0.89 (0.83-0.94)	<.001	0.92 (0.87-0.96)	<.001
Type of loss: relationship with the patient (vs others)						
Spouse	0.62 (0.16-2.31)	.47	0.09 (0.01-0.53)	.01	0.36 (0.05-2.76)	.33
Child	0.19 (0.04-0.95)	.04	0.63 (0.08-5.03)	.67	0.36 (0.04-2.95)	.34
Death-related factors (patient demographics and disease characteristics)						
Age	1.02 (0.98-1.07)	.35	1.02 (0.96-1.08)	.54	0.98 (0.92-1.04)	.53
Gender (men vs women)	0.55 (0.21-1.42)	.22	0.66 (0.23-1.93)	.45	0.36 (0.10-1.32)	.12
Diagnosis (cancer vs noncancer)	1.92 (0.79-4.64)	.15	1.35 (0.45-4.12)	.59	1.57 (0.37-6.66)	.54
APACHE II score	0.95 (0.88-1.02)	.14	0.94 (0.85-1.03)	.17	0.97 (0.85-1.11)	.70
Length of ICU stay	0.98 (0.95-1.02)	.38	0.95 (0.91-1.00)	.04	0.96 (0.90-1.01)	.14
Death circumstances						
Medical record–derived process-based quality indicators of high-quality end-of-life care in ICUs (presence vs absence)						
Prognostic communication	0.32 (0.08-1.29)	.11	4.46 (0.17-117.69)	.37	0.54 (0.05-6.06)	.61
Family meetings conducted	1.58 (0.38-6.57)	.53	8.61 (2.49-29.74)	<.001	7.28 (1.37-38.71)	.02
Palliative care provided	1.50 (0.46-4.88)	.50	0.28 (0.07-1.09)	.07	4.49 (0.68-29.56)	.12
Social worker involvement	2.75 (0.49-15.44)	.25	0.76 (0.09-6.17)	.80	1.58 (0.16-15.27)	.69
Do-not-resuscitate order issued	NA	NA	0.42 (0.04-4.33)	.47	1.89 (0.15-24.12)	.63
Death with cardiopulmonary resuscitation	1.50 (0.17-13.53)	.72	0.34 (0.03-3.91)	.38	7.51 (1.12-50.25)	.04
Withdrawal of life-sustaining treatments	0.76 (0.25-2.27)	.62	0.45 (0.10-2.00)	.29	0.20 (0.02-2.13)	.18
Family presence at patient’s death	0.16 (0.05-0.54)	<.001	0.21 (0.05-0.82)	.03	0.08 (0.02-0.38)	<.001
Family satisfaction						
ICU care	0.97 (0.92-1.01)	.16	0.93 (0.87-0.98)	.01	0.86 (0.81-0.92)	<.001
Decision-making process	1.00 (0.95-1.05)	.93	0.96 (0.89-1.04)	.35	0.94 (0.86-1.02)	.14

Abbreviations: aOR, adjusted odds ratio, APACHE II, Acute Physiology and Chronic Health Evaluation; ICU, intensive care unit; NA, not applicable.

^a^ The high quality-of-death-and-dying class was the reference group.

^b^ In the year before the patients’ critical illness.

Among interpersonal factors, higher surrogate-perceived social support was negatively associated with the poor to uncertain QODD class (aOR, 0.89; 95% CI, 0.83-0.94) and worst QODD class (aOR, 0.92; 95% CI, 0.87-0.96) (Table 3 and the eFigure in Supplement 1). Spousal surrogates were less likely than other surrogates to report patient membership in the poor to uncertain QODD class (aOR, 0.09; 95% CI, 0.01-0.53), and adult-child surrogates were less likely than other surrogates to report patient membership in the moderate QODD class (aOR, 0.19; 95% CI, 0.04-0.95).

Death-related factors indicated by patient demographics and clinical characteristics were not associated with QODD class membership, except for length of stay (Table 3 and the eFigure in Supplement 1). The longer the ICU stay, the lower patient membership in the poor to uncertain QODD class (aOR, 0.95; 95% CI, 0.91-1.00).

Death circumstances measured by process-based indicators of high-quality EOL ICU care and family satisfaction with ICU care were associated with QODD class membership (Table 3 and the eFigure in Supplement 1). Conducting a family meeting before the patient’s death was associated with patient membership in the poor to uncertain (aOR, 8.61; 95% CI, 2.49-29.74) and worst (aOR, 7.28; 95% CI, 1.37-38.71) QODD classes. Death with CPR was associated with the worst QODD class (aOR, 7.51; 95% CI, 1.12-50.25). Family presence at patient death was uniformly negatively associated with the moderate QODD class (aOR, 0.16; 95% CI, 0.05-0.54), poor to uncertain QODD class (aOR, 0.21; 95% CI, 0.05-0.82), and worst QODD class (aOR, 0.08; 95% CI, 0.02-0.38). Higher family satisfaction with ICU care was negatively associated with the poor to uncertain QODD class (aOR, 0.93; 95% CI, 0.87-0.98) and worst QODD class (aOR, 0.86; 95% CI, 0.81-0.92), whereas family satisfaction with EOL-care decision-making process was not associated with QODD class.

## Discussion

Our cohort study comprehensively investigated factors of critically ill patients’ membership in 4 QODD classes previously determined by bereaved family surrogate evaluation. Consistent with the majority of previous reports, we found no patient demographics,^12,21,25,26^ few family demographics,^12,25,27^ scarce patient clinical characteristics,^12,21,25,26,27^ and no incidents of family preexisting psychological distress^28^ associated with family surrogates’ evaluation of their loved one’s quality of dying and death in ICUs. However, death circumstances measured by indicators of care documented in the medical record and bereaved family surrogate satisfaction with EOL ICU care were associated with patient membership in the QODD classes.

We found that the older the family surrogates, the lower the likelihood of patient membership in the worst QODD class relative to the high QODD class. This result is consistent with reports that the older the family member surrogate, the higher the QODD scores assessed for non-ICU decedents from the US,^24^ Canada,^25^ and Israel^27^ and the indirect finding that the oldest US ICU decedents (≥65 years old) had the highest QODD scores.^21^ Most family surrogates in our study were spouses or adult children of the patients (Table 2), thereby older surrogates tended to represent older patients. In Taiwan, deaths of older patients (*shou zhong zheng qin*) are more accepted by patients themselves and their family members as a natural death in contrast to deaths of younger people (*yin nian zao shi*) which are recognized as untimely. Therefore, older patients may be more emotionally prepared for their forthcoming death, consistent with findings^21^ that family members of the oldest ICU decedents rated the domains of preparation for death (eg, saying goodbye to loved ones and funeral arrangements) and connectedness (time spent with family) significantly higher. Furthermore, in accordance with the most fundamental moral duty in Confucianism outlined in the *Xiao Jing* (Book of Filial Duty), children should reciprocate for the life and gracious care they received in their youth through respectful care that physically nourishes their parents and preserves their human dignity.^40^ When LSTs can provide limited benefit and cause pain and discomfort, surrogates of older Taiwanese patients may withhold LSTs and focus on comfort-oriented care to avoid needless protraction of the dying process and let nature take its course,^41^ thereby reducing likelihood of patient membership in the worst QODD class. However, why critically ill patients of surrogates who were men were less likely to be in the poor to uncertain QODD class warrants further investigation, preferably by in-depth qualitative research in light of no associations observed for caregiver gender^12,24,25,26^ with QODD scores.

Among patient demographics and clinical characteristics, only length of ICU stay was associated with QODD class membership. The longer the ICU stay, the lower the patients’ likelihood of being in the poor to uncertain QODD class contrary to no association with total QODD scores reported.^12,25^ Longer ICU stays may give patients and surrogates more time to prepare for and come to terms with the patient’s forthcoming death, thereby decreasing patients’ likelihood of being in the poor to uncertain QODD class.

Among interpersonal risk factors, we found, to our knowledge, a never-before-examined result that surrogates’ higher perceived social support was negatively associated with the poor to uncertain and worst QODD classes. Our results confirm the buffering model that connection, integration, and availability of interpersonal resources in a social network buffer the adverse effects of stressful events^42^ and reduce distress from coping with the imminent loss of a loved one, leading to more favorable perception of QODD of their loved one.

Spousal loss was negatively associated with the poor to uncertain QODD class. It has been shown that the longer the family member has known the decedent, the higher the rating of quality of dying,^12.26^ and spousal caregivers reported higher scores on the whole person domain of the QODD.^27^ Spouses losing a lifelong intimate relationship may feel more confident in appraising whether the patient felt at peace and unafraid of dying, cleared up bad feelings, and said goodbye to loved ones. Spouses may also more aptly make EOL decisions against aggressive LSTs, concordant with common patient preferences,^7,43^ thereby decreasing patient membership in the poor to uncertain QODD class.

Patients with adult-child surrogates had a lower likelihood of moderate than high QODD class membership, although no association with QODD scores was reported.^26^ Adult-child surrogates might actively seek better symptom management for their parent. In Asian countries (like Taiwan), based on the doctrine of filial piety,^40^ adult children (especially the oldest son) are expected to protect their parents from dangers or threats, like severe symptoms from pain or dyspnea, and may more knowledgably advocate for treatments that relieve their dying parent’s suffering than surrogates with other relationships (eg, siblings or other relatives), thereby increasing likelihood for the high QODD class.

Several death circumstances measured by process-based indicators of high-quality EOL care and bereaved family surrogates’ satisfaction with EOL ICU care were associated with patients’ QODD class membership. However, except for death with CPR, restraint of high-intensity LSTs (withdrawal of LSTs or a do-not-resuscitate order issued before death) was not associated with patients’ QODD class membership, consistent with no observed association of intensity of EOL care with QODD scores.^12,24^ Nonbeneficial CPR was associated with the worst QODD class, as indicated in the discussion of filial duty.^40^ Despite health care professional societies’ opposition to nonbeneficial, inappropriate LSTs at EOL,^11,44^ surrogate decision-makers may see treatment appropriateness differently than ICU health care professionals.^45^ Subsequently witnessing implementation of nonbeneficial CPR seems to strongly shape how surrogates appraise their loved one’s QODD. Therefore, EOL-care discussions must gauge the goals and preferences and address the knowledge limitations of patients and their surrogates to facilitate informed, goal-concordant EOL care decision-making.

A documented family meeting was associated with increased membership in the poor to uncertain and worst QODD classes contrary to no observed association with QODD scores.^12^ Interdisciplinary family meetings inform and support ICU family members but are time consuming and resource intensive.^46^ Family meetings are not mandated in Taiwanese ICUs, but Taiwan’s Bureau of Health Insurance reimburses formal interdisciplinary meetings for families in need. We speculate that patients with difficult medical, psychological, and family situations and families facing challenging and complicated EOL-care decisions are targeted for family meetings. These exacerbating circumstances may explain surrogates’ poorer appraisals of QODD among patients in the poor to uncertain and worst QODD classes.

In contrast, family presence at patient death was uniformly negatively associated with the 3 worst QODD classes relative to the high QODD class. Family presence at the time of death was not associated with^12^ or was positively^47^ associated with higher QODD scores. Family presence in the ICU is an evidence-based, family-centered care recommendation.^15^ Our results confirm the benefit of family presence in ICUs,^15^ evident from enhanced quality of dying and death. Indeed, facilitating closure of a longstanding relationship through family presence at the patient’s last moments is an exercise of filial duty in Taiwan.^40^

Higher family satisfaction with quality, continuity, and accessibility of care provided in ICUs was negatively associated with the 2 worst QODD classes. Higher QODD scores were associated with better symptom management, availability of a health care team member to ensure patients the best care, physician-clinician communication about treatment preferences, care aligned with treatment preferences, and satisfaction with communication.^24^ Patient comfort and communication are the 2 processes of ICU care that matter most to patients and their families.^48^ Our results suggest that bereaved surrogates’ higher satisfaction with EOL care provided in ICUs (eg, adequate symptom management and appropriate communication) may reflect that their loved one died comfortably without pain and suffering; had a dignified, emotionally peaceful, fearless, and prepared death; and received fewer goal-discordant LSTs, thereby increasing the patient’s likelihood of being in the high QODD class.

### Limitations

This study has limitations. We sampled from 2 hospitals in Taiwan, limiting generalizability to national and international populations. We cannot generalize results to family members of ICU patients who died within 3 days of admission, or to those who did not participate in bereavement surveys. Validity of the 4 previously determined QODD classes warrants confirmation. Concurrently measuring perceived social support and family satisfaction with EOL ICU care alongside evaluation of the patient’s quality of dying and death may have bilaterally colored responses, but we adopted this approach for feasibility. A causal relationship between factors and QODD classes cannot be inferred in this observational study, nor can the possibility of unmeasured covariates be excluded (eg, physician-surrogate agreement on appropriateness of treatment and surrogates’ preparedness for death and their coping strategies).

## Conclusions

In this cohort study of critically ill patients and their family surrogates, modifiable EOL ICU care played a more significant role in associations with critically ill patients’ QODD class than immutable family demographics, preexisting family health conditions, patient demographics, and patient clinical characteristics. Actionable EOL-care interventions to achieve high-quality dying and death for critically ill ICU patients include leveraging psychological, social, and informational support for the dying patient and their surrogate in difficult and complicated situations through family meetings, avoiding nonbeneficial CPR, facilitating family social support and presence at patient death, and enhancing family satisfaction with care by providing adequate symptom management and appropriate communication.

## Notes

Supplement 1. **eMethods.** Identification of the Four QODD Classes by Latent Class Analysis
eReferences.
**eFigure.** Graphic of Factors of Quality-of-Dying-and-Death Classes Perceived by Bereaved Family Surrogates of Critically Ill Patients in ICUs

Supplement 2.
Data Sharing Statement
