# Supplementary material for: Factors Associated With Quality-of-Dying-and-Death Classes Among Critically Ill Patients
Source: JAMA Netw Open. 2024 Jul 1;7(7):e2420388. doi: 10.1001/jamanetworkopen.2024.20388 (PMC11217872; doi:10.1001/jamanetworkopen.2024.20388)
Supplement: Supplement 1. — eMethods. Identification of the Four QODD Classes by Latent Class Analysis eReferences. eFigure. Graphic of Factors of Quality-of-Dying-and-Death Classes Perceived by Bereaved Family Surrogates of Critically Ill Patients in ICUs [file jamanetwopen-e2420388-s001.pdf]

Supplemental Online Content

Wen FH, Chou WC, Huang CC, Hu TH, Chuang LP, Tang ST. Factors associated with quality of dying and death classes among critically ill patients. *JAMA Netw. Open*. 2024;7(7):e2420388. doi:10.1001/jamanetworkopen.2024.20388

eMethods. Identification of the Four QODD Classes by Latent Class Analysis
eReferences
eFigure 1. Graphic of Factors of Quality-of-Dying-and-Death Classes Perceived by Bereaved Family Surrogates of Critically Ill Patients in ICUs

This supplemental material has been provided by the authors to give readers additional information about their work.

© 2024 Wen FH et al. *JAMA Network Open.*

eMethods. Identification of the Four QODD Classes by Latent Class Analysis

We retained the items with a response of "don't know" and dichotomized the 6-category responses of the frequency component into none/a little bit/some/a good bit of the time vs most/all of the time[1,2] for identifying patterns of bereaved surrogates' evaluation of their relative's quality of dying and death using latent class analysis (LCA) by Latent GOLD 5.1. In this approach, evaluations of quality of dying and death were treated as a pattern or set of QODD items ("latent class") rather than individual items.[3] LCA divides bereaved surrogates into mutually exclusive probabilistic classes based on shared characteristics that discriminate among members of each class. LCA simultaneously estimates class probabilities (the number and relative size of classes) and conditional probabilities across classes. Conditional probability reflects the probability that a surrogate in a given class experienced that QODD item none/a little bit/some/a good bit of the time, most/all of the time, or unknown.[3]

Best model solutions with an increasing number of classes were selected by model fit indices, including several information criteria (IC), i.e., the highest log-likelihood, smallest Akaike (AIC), consistent AIC (CAIC), Bayesian (BIC), and sample-size adjusted BIC (SABIC).[4] Whereas the BIC is recognized as the best of the IC, the flattening of IC values between consecutive numbers of hidden classes in plots of IC values *vs*. class number suggests that the increase in class number is not meaningful.[3,4] These criteria, parsimony, and the clinical

© 2024 Wen FH et al. *JAMA Network Open.*

meaningfulness of the latent class identification, were factored in deciding the optimal number of classes.

Three items without variances (99.0-100.0%) across participants were deleted for LCA. The four-class solution was selected as optimal due to the best combination of ICs (giving a higher priority to the plots of ICs values), parsimony, and clinical meaningfulness.

eReferences.

1. Levy CR, Ely EW, Payne K, Engelberg RA, Patrick DL, Curtis JR. Quality of dying and death in two medical ICUs: perceptions of family and clinicians. Chest 2005;127:1775-1783.
2. Mularski RA, Heine CE, Osborne ML, Ganzini L, Curtis JR. Quality of dying in the ICU: ratings by family members. Chest 2005;128:280-287.
3. Muthén B. Latent variable analysis: Growth mixture modeling and related techniques for longitudinal data. In: Kaplan D, editor. The Sage handbook of quantitative methodology for the social sciences. Thousand Oaks, CA: Sage; 2004. pp. 345–369.
4. Geiser C. Data analysis with Mplus. 2012. New York City: Guilford Press.

© 2024 Wen FH et al. *JAMA Network Open.*

eFigure. Graphic of Factors of Quality-of-Dying-and-Death Classes Perceived by Bereaved Family Surrogates of Critically Ill Patients in ICUs

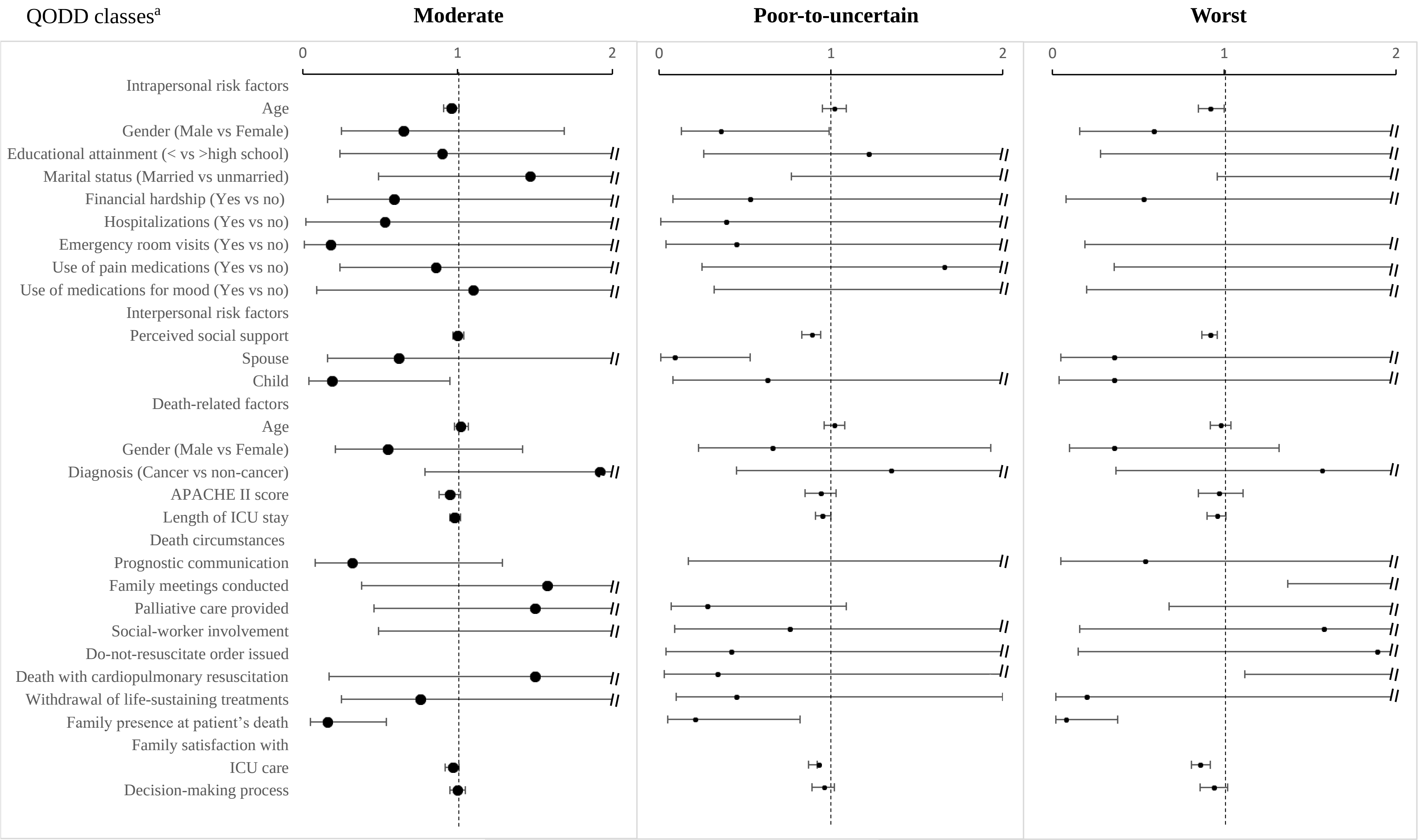

a: in reference to high QODD class; "//" indicates the upper limit of 95%CI >2.00

© 2024 Wen FH et al. *JAMA Network Open.*
