# Supplementary material for: Factors Associated With Quality-of-Dying-and-Death Classes Among Critically Ill Patients
Source: JAMA Netw Open. 2024 Jul 1;7(7):e2420388. doi: 10.1001/jamanetworkopen.2024.20388 (PMC11217872; doi:10.1001/jamanetworkopen.2024.20388)
Supplement: Supplement 2. — Data Sharing Statement [file jamanetwopen-e2420388-s002.pdf]

Data Sharing Statement

Wen. Factors Associated With Quality-of-Dying-and-Death Classes Among Critically Ill Patients. *JAMA Netw Open*. Published July 01, 2024.
doi:10.1001/jamanetworkopen.2024.20388

Data
Data available: No

Additional Information
Explanation for why data not available: The sharing of anonymized data from this study is restricted due to ethical and legal constrictions. Data contains sensitive personal health information, which is protected under The Personal Data Protection Act in Taiwan, thus making all data requests subject to Institutional Review Board (IRB) approval. Per Chang Gung Memorial Hospital (CGMH) IRB, the data that support the findings of this study are restricted for transmission to those in the primary investigative team. Data sharing with investigators outside the team requires IRB approval. All requests for anonymized data will be reviewed by the research team and then submitted to the CGMH IRB for approval. Upon approval from the Chang Gung Medical Foundation IRB, the data supporting the findings of this study are available from the corresponding author (Dr Siew Tzuh Tang) upon reasonable request.
